# Surface Characterization, Biocompatibility and Antifungal Efficacy of a Denture-Lining Material Containing *Cnidium officinale* Extracts

**DOI:** 10.3390/molecules26051440

**Published:** 2021-03-07

**Authors:** Myung-Jin Lee, Youn-Soo Shim, So-Youn An, Min-Kyung Kang

**Affiliations:** 1 Department of Dental Hygiene, Division of Health Science, Baekseok University, Cheonan 31065, Chungcheongnam-do, Korea; dh.mjlee@bu.ac.kr; 2 Department of Dental Hygiene, Sunmoon University, Asan 31460, Chungcheongnam-do, Korea; shim-21@hanmail.net; 3 Department of Pediatric Dentristry, College of Dentistry, Wonkwang University, Iksan-si 5453, Jeollabuk-do, Korea; 9543sue@hanmail.net; 4 Wonkwang Bone Regeneration Research Institute, College of Dentistry, Wonkwang University, Iksan-si 5453, Jeollabuk-do, Korea; 5 Department of Dental Hygiene, Hanseo University, Seosan 31963, Chungcheongnam-do, Korea

**Keywords:** antifungal efficacy, biocompatibility, *Candida albicans*, *Cnidium officinale*, denture-relining material, surface characterization

## Abstract

Herein, we investigated the surface characterization and biocompatibility of a denture-lining material containing *Cnidium officinale* extracts and its antifungal efficacy against *Candida albicans*. To achieve this, a denture-lining material containing various concentrations of *C. officinale* extract and a control group without *C. officinale* extract were prepared. The surface characterization and biocompatibility of the samples were investigated. In addition, the antifungal efficacy of the samples on *C. albicans* was investigated using spectrophotometric growth and a LIVE/DEAD assay. The results revealed that there was no significant difference between the biocompatibility of the experimental and control groups (*p* > 0.05). However, there was a significant difference between the antifungal efficiency of the denture material on *C. albicans* and that of the control group (*p* < 0.05), which was confirmed by the LIVE/DEAD assay. These results indicate the promising potential of the *C. officinale* extract-containing denture-lining material as an antifungal dental material.

## 1. Introduction

Soft denture-lining materials are generally used to moisten damaged mucous membranes of existing poorly fitted dentures, or to temporarily fit the tissue surface of a new denture or an existing denture before making a new denture [1]. Commonly used clinical denture-lining materials, such as the mixture of polyethylmethacrylate and an ester-based liquid plasticizer in ethyl alcohol solution without an acrylic monomer, exhibit plasticity and viscoelastic properties [2]. This enables the adhesion of the denture base to the denture support tissue at the beginning of the reaction because of functional stress [1,3]. However, the surface of the denture-lining material becomes hard and rough, as it is partially replaced. Previous studies have reported that this reaction mechanism causes the denture-lining material to rapidly lose its initial viscoelastic properties, which is accompanied by changes in the volume over time [4]. Furthermore, the loss of plasticity causes changes in the physical state of the denture-lining material, such as degeneration depending on the mastication pressure and the elapsed action time [4,5]. In addition, because it is used in direct contact with oral tissues, poor denture management enables the colonization of microorganisms, like *Candida albicans*, on the surface of the denture. This often leads to complications like *Candida*-related denture stomatitis [6,7]. Therefore, preventing the growth of bacterial colonies on dentures through the proper hygiene management of dentures is important. Mechanical and chemical cleaning have been introduced as effective methods to manage denture hygiene. Mechanical methods include the use of a toothbrush, toothpaste and powder, and ultrasonic cleaners, while chemical methods include the use of denture cleaners and disinfectants, such as chlorhexidine, salicylate, and glutaraldehyde [7]. However, these cleaning methods change the physical properties of the material [8,9]. In addition, the application of mechanical methods by inactive elderly patients is difficult. Furthermore, mechanical methods create a microorganism-friendly denture environment by forming abrasions on the denture surface; meanwhile, the chemical method prevents the bleaching action of sodium hypochlorite or the decomposition of the organic polymer structure [7,10,11]. Therefore, developing biostable dentures with antimicrobial properties is important. However, despite the tremendous effort devoted to developing new materials, a biostable material with antimicrobial properties has yet to be achieved.

Recently, several studies have devoted considerable effort to investigating natural products, with antibacterial properties and few side effects, to develop dental materials containing natural antibacterial substances [7,12]. Among the natural alternatives, *Cnidium officinale* (CO), a perennial herb belonging to the umbel family, has been reported to possess excellent antibacterial, antifungal, sedative anti-inflammatory, and analgesic properties [13,14,15]. Several studies have reported the antioxidant and antimicrobial effects of CO extract. However, there are no studies that have investigated the application of CO as a denture-lining material and its antibacterial effect against oral bacteria [14,16].

Therefore, the aim of this study is to investigate the physical and biological properties of denture-lining material containing CO extract and evaluate its antifungal activity against *C. albicans*, the causative agent of dental stomatitis.

## 2. Results and Discussion

Denture-lining materials are widely used to treat damaged mucosal tissues underlying ill-fitting acrylic dentures; however, their poor antifungal efficacy has limited their further application [2,17]. Previous studies have reported that, depending on the surface characteristics, the surface of unpolished dentures causes the coloring of dentures, accumulation of food residues, and the deposition of bacteria [3]. In this study, the surface characterization, cytotoxicity, and antifungal efficacy of denture-lining material containing the CO extract were investigated.

**Figure 1:**
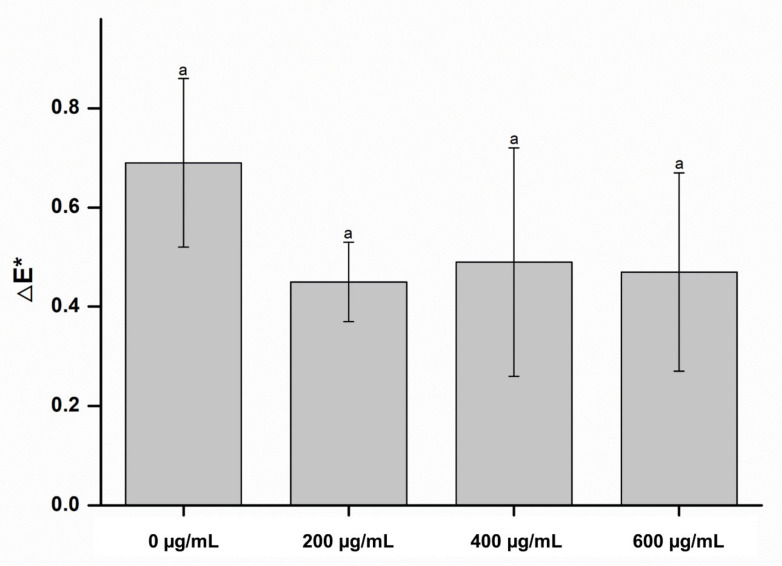
Color change in the experimental and control groups. Each value represents the mean of five measurements, and the error bar shows the standard deviation of the mean value. The lowercase letter indicates that there were no significant differences between the values of the experimental and control groups (*p* > 0.05).

**Figure 2:**
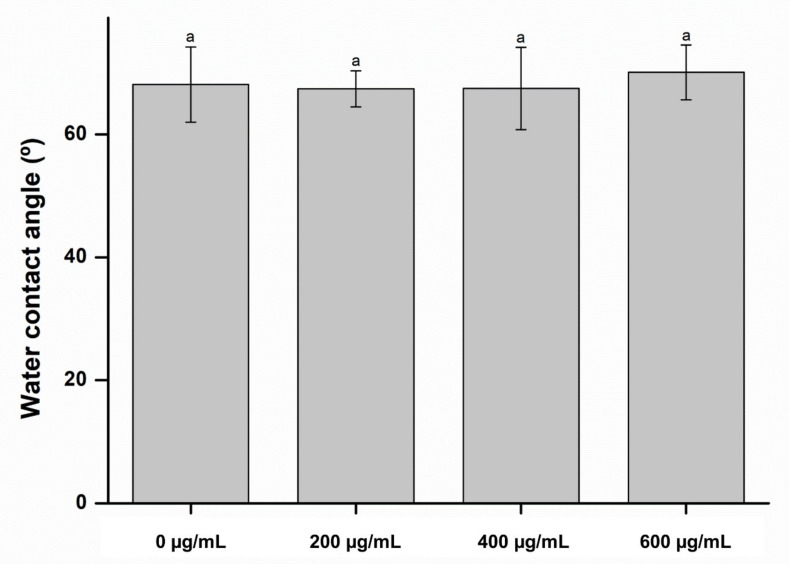
Wettability of the experimental and control groups. Each value represents the mean of five measurements, and the error bar shows the standard deviation of the mean value. The lowercase letter indicates that there were no significant differences between the values of the experimental and control groups (*p* > 0.05).

Surface characterizations, such as color change and wettability, are important characteristics of denture-lining materials [18]. Color stability is a critical clinical requirement for denture-lining material owing to aesthetic reasons. In this study, there were no significant differences between the color change in the experimental groups and control group (*p* > 0.05), indicating that the CO had no significant effect on the color of the denture-lining material, whose results are shown in Figure 1. In addition, there were no significant differences between the wettability of the control and experimental groups (*p* > 0.05), as shown in Figure 2. These results indicate that the denture-lining material containing the CO extract had stable surface characteristics.

**Figure 3:**
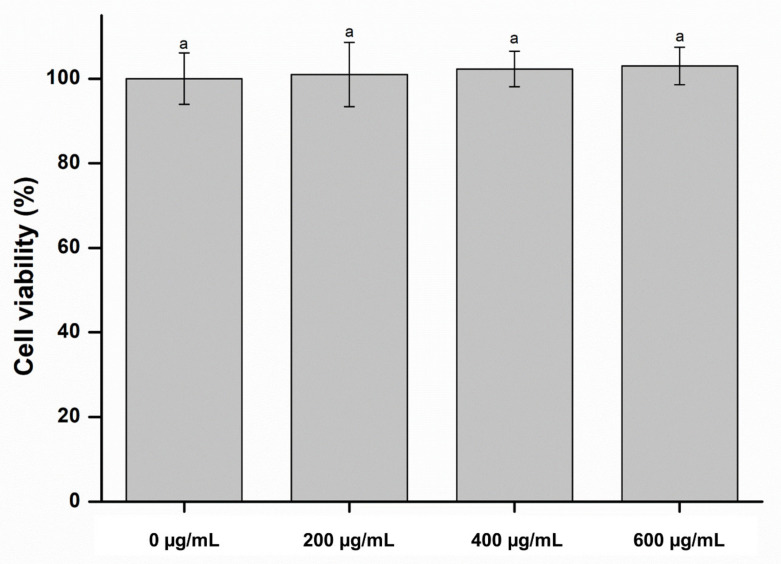
Cell viability of the experimental and control groups. Each value represents the mean of five measurements, and error bar shows the standard deviation of the mean. The lowercase letter indicates that there were no significant differences between the values of the experimental and control groups (*p* > 0.05).

The biological properties of the denture-lining materials are also important for clinical application [6]. As denture-lining materials are in direct contact with the oral mucosa, investigating the cytotoxicity of the denture-lining material is important [4,6,19]. The in vitro biological evaluation of clinical materials is exceedingly crucial [20]. Therefore, an MTT assay was carried out on the denture-lining material containing CO to simulate the oral cavity to assess its cytotoxicity. There was no significant difference between the cell viability of the control group and that of the experimental groups (*p* > 0.05), as shown in Figure 3. This result indicates that the denture-lining material containing the CO extract exhibited no cytotoxicity.

Over 500 types of microorganisms, including bacteria, fungi, and viruses survive in the oral cavity in the form of a colony known as a microbiota [21,22,23]. In particular, the presence of an abundance of the fungus *C. albicans* in the oral cavity causes infections, collectively known as candidiasis [7,24]. 

**Figure 4:**
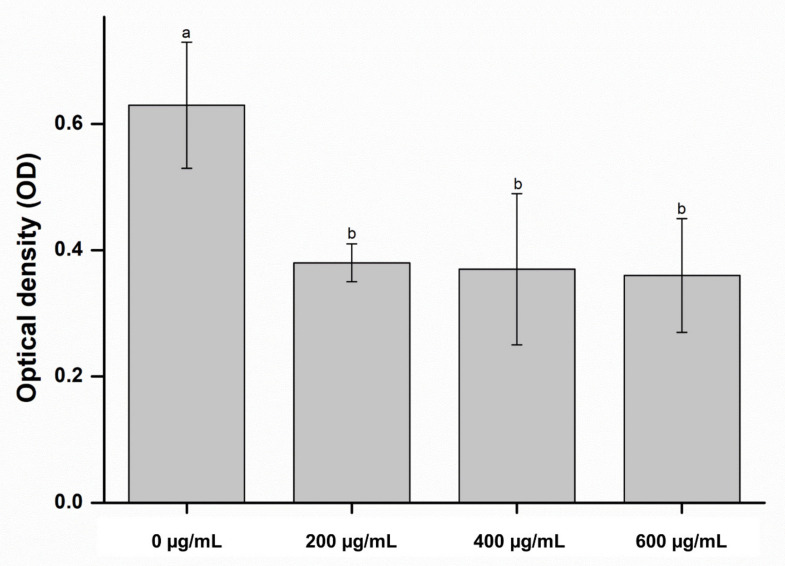
Optical density of the experimental and control groups. Each value represents the mean of five measurements, and the error bar shows the standard deviation of the mean value. The same lowercase letter indicates that there were no significant differences between the values of the experimental and control groups (*p* > 0.05).

**Figure 5:**
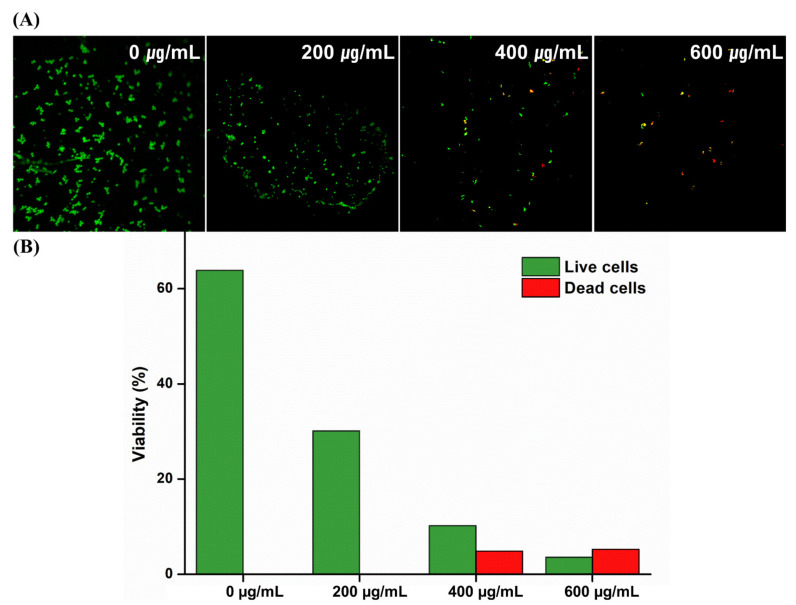
(**A**) Representative microscopic images of the *C. albicans* adhered on the surfaces of the denture-lining material: 0, 200, 400, and 600 µg/mL at a magnification of 5000×, (**B**) quantified fungal viability results of the live and dead cells in equivalent surface areas.

Figure 4 shows the microbial results of the antifungal assay. The OD of *C. albicans* significantly decreased in samples containing CO extracts (*p* < 0.05). In addition, these findings were confirmed by fungal viability staining results, which showed substantially fewer live fungi on the surfaces of the soft denture-lining material with the CO extract than on the control material (Figure 5).

Additionally, the fungal survival rate confirmed that the absorbance decreased with an increase in the concentration of the extract. The decrease in absorbance indicates a decrease in the activity of the fungi. In Figure 5, the fungal viability was calculated by using Image J software to show green fluorescence compared to the area. Since fungi were not attached to the entire area, the viability seemed to exceed 60%. However, it was confirmed that all the attached fungi were alive and there were no dead fungi. The findings of this study indicate that the CO extract improved the antifungal efficacy of denture-lining material without significantly affecting the physical and biological properties of the surface of the material. 

However, the mechanism of the antifungal effect of CO was not completely understood. Previous studies have attributed the antifungal properties of CO to phenolic compounds, which are reference substances that exhibit antibacterial activity in natural extracts [13]. Furthermore, studies have reported on the antibacterial properties of phenolic compounds, such as polyphenol and flavonoids, against *C. albicans* [12,25]. The antioxidant activity and effect of the phenolic component of CO were confirmed by previous studies, along with its antibacterial activity against *Streptococcus mutans* and *C. albicans*, which are two major oral pathogens [21,26]. Previous studies have demonstrated that CO were established, as well as secondary metabolites, such as phenolic, flavonoid, phthalide, and 3-butylidenephthalide synthesis. They also inhibited nitric oxide synthase and cyclooxygenase-2 mRNA expression induced by lipopolysaccharide [16]. The results of this study are limited to the antifungal efficacy of CO, and there is a limitation that the antifungal effect has not been demonstrated through comparative studies with standard antifungal agents. Nevertheless, this study is the first to evaluate the antifungal efficacy of denture-lining material containing CO extract. Therefore, further studies using standard antifungal agents are required to investigate the effect that the CO extract has on various strains. In addition, long-term observations and in vivo tests are needed to clarify the antifungal mechanism of CO for clinical applications.

## 3. Materials and Methods

### 3.1. Extraction

*C. officinale* (purity 99.7%), planted in the Sobaek Mountains placed in Gyeongsangnam-do, South Korea, was commercially purchased. The purchased CO was compared to the standard herbal sample supplied by the National Institute of Food and Drug Safety Evaluation. To obtain the CO extract, 500 g of the CO leaves was crushed and placed in a solution of 70% methanol and extracted at (25 ± 1 °C) for 24 h. The extracts were initially filtered (Filter paper #2, Whatman, Maidsone, U.K.), following which the filtered extract was concentrated using a vacuum evaporator (Vacuum Evaporator, ETELA, Tokyo, Japan). Subsequently, the concentrated extract was pulverized using a freeze-dryer (Freeze Dryer, Ilshin Lab, Kyeonggi-do, Korea) for 48 h. To prepare various concentrations of *C. officinale* extract, the pulverized powder was added to dimethyl sulfoxide (DMSO; Amresco, VWR Life Science, MO, USA) at concentrations of 0, 200, 400, and 600 µg/mL.

### 3.2. Preparation of the Denture-lining Material Containing Cnidium Officinale

Coe-comfort (GC, Japan), a denture-lining material supplied as a powder and liquid was selected for the preparation of the test specimen. To prepare the denture-lining material containing the CO extract, the as-prepared CO powder (200, 400, and 600 μg/mL) was added into the monomer solution. Subsequently, the monomer and the powder were mixed according to the manufacturer’s instructions. The mixture was then poured into a mold (1.0 ± 0.1 mm thick with a diameter of 10.0 ± 0.1 mm) and kept for a fixed time.

### 3.3. Measurement of the Color Change

To evaluate the effect of the CO extract on the color of the denture-lining material, the color change in the samples was investigated using a spectrophotometer (Lamba20, Perkin Elmer, Orwalk, CT, USA) to evaluate the color of the samples. To compare the values before and after the addition of the CO extracts, the color difference was calculated using this equation:

(1) $$\Delta E^{*}=\sqrt{{\left(\Delta L^{*}\right)}^{2}+{\left(\Delta a^{*}\right)}^{2}+{\left(\Delta b^{*}\right)}^{2}}.$$

### 3.4. Measurement of the Wettability

The wettability of the samples was measured based on the contact angle using a contact angle analyzer (Phoenix 300, Surface Electro Optic, Gyeonggi-do, Korea) and contact angle measurement software (Image XP version 5.9, Surface Electro Optics).

### 3.5. Evaluation of Biological Properties

The biological properties of the samples were investigated by performing an MTT (3- [4,5-Dimethylthiazol-2-yl] -2,5-diphenyltetrazolium bromide) assay in accordance with the test method of ISO 10993-5 standards. The number of L929 cells per well, seeded to 1 × 10^4^; 100 μL, was dispensed into the wells and cultured for 24 h. After incubation, 100 μL of the natural extract diluted to various concentrations was applied to the cells for 24 h. As a control, RPMI 1640 (Gibco Laboratories, Grand Island, NY, USA) without natural extract was used. After application, the extract was removed and washed with 100 μL of DPBS (Gibco BRL, Life Technologies, NY, USA). After washing, DPBS was removed, and 50 µL per well was added to 1 mg/mL of MTT (Sigma Chemical Co. Ltd, Poole, UK) and cultured for 2 h. To dissolve the formed MTT formazan, 100 mL of isopropanol (Sigma Chemical Co. Ltd, Poole, UK) was added to the 100 µL/well and incubated for 20 min. The absorbance of the cells was measured at 570 nm on a spectrophotometer and analyzed. The results were normalized to 100% of the MTT reduction rates for the control and experimental groups, expressed in the form of percentages.

### 3.6. Preparation of C. Albicans Strains

For the microbial analysis, *C. albicans* (ATCC 10231) strains were used in the experiment. The *C. albicans* was cultured in a yeast mold medium (Becton Dickinson and Co., Franklin Lakes, NJ, USA) and incubated at 37 °C for 24 h. Additionally, yeast mold medium was used to dilute the culture until the concentration reached nearly 1 × 10^7^ CFU/mL.

### 3.7. Evaluation of Optical Density (OD)

The microbial culture fluid was diluted such that the OD 600 value was between 0.4 and 0.6. Subsequently, the OD of the microbial cultures treated with *C. officinale*/DMSO (0, 200, 400, 600) was measured to verify the microbial viability of the sample. The sample solution and fungi culture were mixed at a ratio of 9:1 and incubated at 37 °C for 24 and 48 h. Using an ELISA plate reader (Epoch, BioTeck, Winooski, VT, USA) at 600 nm, the inhibitory effect of the extracts was determined based on the OD values in each well.

### 3.8. Evaluation of Microbial Viability

To investigate the viability of the *C. albicans*, the samples were stained using a LIVE/DEAD FungaLight Yeast Viability Kit (Molecular Probes, Eugene, OR, USA) according to the manufacturer’s instructions. Equal volumes of SYTO9 dye and propidium iodide from the kit were mixed intravenously. Subsequently, 3 μL of the mixture was added to 1 mL of the as-prepared *C. albicans* suspension. Following this, the mixture was incubated for 15 min at room temperature in the dark, after which the stained samples were observed by confocal laser microscopy (CLSM, LSM880; Carl Zeiss, Thornwood, NY, USA). Live microbes appeared green, while dead microbes appeared red. The live and dead fungi were quantified using the ImageJ software (NIH, Bethesda, MD, USA).

### 3.9. Statistical Analysis

All statistical analyses were conducted using the SPSS 23 software program (IBM Corp., Armonk, NY, USA). The results among different groups were analyzed by a one-way analysis of variance (ANOVA), followed by Tukey’s post hoc test at a significance level of 0.05.

## 4. Conclusions

In this study, we examined the effect that the CO extract had on the surface properties, cytotoxicity, and antifungal efficacy of a denture-lining material. The in vitro study revealed that there was no significant difference between the surface characterization of the experimental group and that of the control group, including the color change, wettability, and biocompatibility (*p* > 0.05). In addition, the denture-lining material containing the CO extract exhibited antimicrobial efficacy against *C. albicans* (*p* < 0.05). Therefore, the results of this study exhibit that the soft, denture-lining material containing CO extract is a promising antimicrobial denture-lining material for the prevention of oral stomatitis.

## Data Availability

Not applicable.
